# Vitamin D status is associated with skin autofluorescence in patients with type 2 diabetes mellitus: a preliminary report

**DOI:** 10.1186/s12933-015-0250-z

**Published:** 2015-07-16

**Authors:** Y H M Krul-Poel, R Agca, P Lips, H van Wijland, F Stam, S Simsek

**Affiliations:** Department of Internal Medicine, Medical Centre Alkmaar, PO Box 7057, Wilhelminalaan 12, 1815 JD Alkmaar, The Netherlands; Department of Internal Medicine/Endocrinology, VU University Medical Centre, Amsterdam, The Netherlands; Department of General Practice, DIAZON, Alkmaar, The Netherlands

**Keywords:** Type 2 diabetes mellitus, Advanced glycation endproducts, Vitamin D, Vitamin D deficiency

## Abstract

**Background:**

Skin autofluorescence is a non-invasive measurement of advanced glycation end products (AGE), which are suggested to be one of the major agents in the pathogenesis and progression of diabetes related cardiovascular complications. Recently, low vitamin D status has been linked to the progression of type 2 diabetes mellitus (T2DM) and cardiovascular disease. The aim of this study is to investigate the association between vitamin D status and skin autofluorescence in patients with T2DM.

**Methods:**

In this preliminary report skin autofluorescence was measured non-invasively with an AGE-reader in 245 patients with T2DM treated with lifestyle advice, metformin and/or sulphonylurea-derivatives. All patients were randomly assigned to receive either vitamin D 50,000 IU/month or placebo for 6 months.

**Results:**

Skin autofluorescence was significantly higher in patients with a serum 25(OH)D <50 nmol/l compared to patients with a serum 25(OH)D >75 nmol/l (2.81 versus 2.41; p < 0.001). Mean serum 25(OH)D was 60.3 ± 23.4 nmol/l and was independently associated with skin autofluorescence (β −0.006; p < 0.001). Mean vitamin D increased from 60.8 to 103.6 nmol/l in the intervention group, however no effect was seen on accumulation of skin AGEs after 6 months compared to placebo.

**Conclusions:**

Vitamin D status is independently associated with skin auto fluorescence in patients with well-controlled T2DM. No effect was seen on the amount of skin AGEs after a short period of 6 months vitamin D supplementation. Further research with longer follow-up and measurement of circulating advanced glycation end products is needed to elucidate the causality of the association.

**Electronic supplementary material:**

The online version of this article (doi:10.1186/s12933-015-0250-z) contains supplementary material, which is available to authorized users.

## Background

One of the chronic consequences of hyperglycaemia is the accelerated formation of advanced glycation end products (AGEs), which are suggested as one of the major pathogenic mechanisms causing end organ damage in diabetes [1, 2]. AGEs are formed nonenzymatically by the modification of proteins, lipids and nucleic acids by glucose. AGEs are highly reactive pro-inflammatory molecules and accumulate slowly over a persons’ lifetime with an estimated lifetime of 15 years [3, 4]. Diabetes mellitus, chronic renal failure and aging accelerate the generation of AGEs [5]. AGEs act by binding to the receptor for AGEs (RAGE), which is expressed on neutrophils, T-lymphocytes, macrophages and synovial fibroblasts. Upon binding of AGE to its receptor the transcription of pro-inflammatory genes is stimulated, leading to the up-regulation of endothelial adhesion molecules contributing to the development of atherosclerosis [6]. In patients with type 2 diabetes mellitus (T2DM) AGEs seem to represent inflammatory, oxidative and cumulative metabolic stress [3]. Earlier research demonstrated an increased skin autofluorescence, as a measure of AGE accumulation, in patients with T2DM compared to healthy controls [7]. In addition, skin autofluorescence predicts both micro- and macrovascular complications in patients with T2DM, and has a strong association with the severity of diabetes-related complications and mortality [2, 8–14].

Similar to the consequences of AGE accumulation, low vitamin D status has been linked to numerous biochemical and clinical disturbances, including the pathogenesis and progression of T2DM and cardiovascular disease [15–18]. The prevalence of vitamin D deficiency is high worldwide, especially in patients with T2DM compared to healthy persons [19, 20]. The underlying mechanism explaining the association between vitamin D status, T2DM and cardiovascular disease is not clarified yet. Earlier data suggest a potential independent role for vitamin D in the regulation of glucose metabolism in a setting of obese patients [21]. It is known that besides the classical role of vitamin D in calcium and bone homeostasis, vitamin D is linked to numerous non-skeletal diseases due to the elucidation that most cells, including the pancreatic beta-cells and cardiomyocytes contain the vitamin D receptor (VDR), and most of them also have the capability to produce the biologically active 1,25-dihydroxyvitamin D for paracrine functions [22, 23]. Experimental studies have established a role for vitamin D in T2DM and cardiovascular disease by interacting with inflammation, insulin resistance, renin-angiotensin system, thrombosis and gene regulation [24–28]. To our knowledge no literature is available about the association between AGEs and vitamin D status in patients with T2DM. Hypothetically, vitamin D may reduce inflammation, oxidative stress and insulin resistance, and thereby reducing the accumulation of AGEs. Interestingly, in diabetic rats vitamin D supplementation attenuated the deposition of AGEs in the vascular system [29] and decreased the diabetic effects on the receptor of AGEs in rats [30]. Therefore, the aim of our study is to investigate the association between vitamin D status and skin auto fluorescence in patients with T2DM.

## Methods

We performed a preliminary cross-sectional and longitudinal analysis of 245 patients with T2DM. The patients were included between July 2012 and April 2013 in a randomised placebo-controlled trial (“*the SUNNY trial*”), in which the effect of 50,000 IU/month vitamin D supplementation during 6 months versus similar looking placebo was examined on glycemic control in patients with T2DM [31]. The trial was approved by the Medical Ethics Committee of North-Holland, the Netherlands. The trial protocol is described in greater detail elsewhere [32]. In brief, adult patients (≥18 years), treated with lifestyle advice, metformin or sulphonylurea-derivatives, whether or not in combination, were invited for participation in the trial. The main exclusion criteria were previous treatment with insulin, serum 25-hydroxyvitamin D (25(OH)D) <15 nmol/l or >150 nmol/l, hypercalcaemia (serum calcium >2.65 nmol/l), impaired renal function (estimated glomerular filtration rate [eGFR] <30 ml/min, measured by the MDRD formula) urolithiasis, and no signed informed consent. Throughout the study no drug alterations regarding hypoglycemic agents and statins, and no vitamin D supplements were allowed. Informed consent was obtained from all patients before start of the trial.

### Outcome measures

Outcome measures were obtained at baseline (immediately prior to dosing), and after 6 months. The following data were collected during the first visit: age, gender, ethnicity, social status, diabetes duration, medical history, family history of T2DM, medication use, diabetic complications, previous cardiovascular disease, co-morbidity, smoking status, alcohol use, diet (especially fish and dairy products), physical activity, sun exposure, and season of blood collection. Cardiovascular disease was defined as coronary artery disease/ischemic heart disease, stroke, hypertensive heart disease, aortic aneurysms, cardiomyopathy and peripheral artery disease. Standard anthropometric data (height, weight, waist and hip circumference, blood pressure) were obtained from each patient. Venous blood collection for serum 25(OH)D, HbA_1c_, fasting blood glucose and insulin, lipid profile, serum calcium, albumin, estimated glomerular filtration rate (eGFR) and parathyroid hormone (PTH) were collected after an overnight fast at 8.00–9.30 am. Serum 25(OH)D was measured on an iSYS automated immunoanalyser (IDS GmbH, Frankfurt, Germany). All samples are stored at −20°C for future research on circulating AGE levels.

### Skin autofluorescence

Skin autofluorescence levels were measured with an AGE reader (Diagnoptics Technologies B.V., Groningen, the Netherlands). This non-invasive method utilizes the characteristic fluorescent properties of AGEs, and has been validated with specific AGE measurements in skin biopsies [33]. Autofluorescence was defined as the average light intensity per nanometer in the range between 420 and 600 nm, divided by the average light intensity per nanometer in the range between 300 and 420 nm. It was expressed in arbitrary units (AU) using the AGE reader software. Skin autofluorescence was measured at room temperature while patients were in a seated position, at the volar side of the lower arm. Measurements were not specifically performed in a fasting state. Previous research has demonstrated that repeated AGE measurements on 1 day showed an overall Altman error percentage <6.0%. Intra-individual seasonal variance showed an Altman error percentage <6.0% [33].

### Statistical analysis

All data were analysed using the statistical package SPSS software (version 20.0, SPSS Inc, Chicago, IL, USA). For the purpose of our study and consistent with the widely used cut-off values of vitamin D, the patients were stratified into three vitamin D groups: (1) serum 25(OH)D: 15–49 nmol/l, (2) serum 25(OH)D: 50–74 nmol/l, and (3) serum 25(OH)D: 75–150 nmol/l [34]. Baseline characteristics were compared using χ^2^-test for categorical variables and ANOVA or Kruskal–Wallis for continuous variables depending on normal distribution. Data are presented as mean ± standard deviation if normally distributed and otherwise as median and interquartile range. Multiple linear regression analysis was used to determine independent associations between vitamin D status and skin autofluorescence. We adjusted for confounders based on the literature and regression correlation coefficient difference >10%: age, season of measurement, duration of diabetes, renal function, gender, ethnicity, smoking behaviour, lipid profile, HbA_1c_, and the presence of cardiovascular disease. Linear regression analysis was used to assess the mean difference between intervention and placebo groups after 6 months (mean difference is reported as beta). A *p* value <0.05 was considered as statistically significant.

## Results

Baseline skin autofluorescence level was determined in 245 of 275 patients included in the SUNNY trial. Skin autofluorescence was not measurable in 30 patients, mainly due to low reflection caused by dark coloured skin. Demographic, anthropometric and clinical characteristics of all 245 patients, and stratified to vitamin D level are presented in Table 1. The mean age of the patients was 67 ± 8 years and 64% were male, with a median diabetes duration of 6.0 (3.0–8.0) years. Overall mean serum 25(OH)D was 60.3 ± 23.4 nmol/l and mean skin autofluorescence 2.64 ± 0.6. Vitamin D deficiency (serum 25(OH)D <50 nmol/l) was present in 89 patients (37%), 96 patients (39%) had a serum 25(OH)D level between 50–74 nmol/l and 60 patients (24%) had a serum 25(OH)D >75 nmol/l.

**Table 1:** Baseline demographic and clinical characteristics

	All	Serum 25 (OH)D 15–49 nmol/l	Serum 25 (OH)D 50–74 nmol/l	Serum 25 (OH)D 75–150 nmol/l	*p* value
N	245	89	96	60	
Male (%)	156 (63)	49 (55)	66 (69)	41 (68)	0.09
Age (years)	67 ± 8	67 ± 9	67 ± 9	67 ± 7	0.82
White skin colour (%)	234 (95)	80 (90)	93 (97)	60 (100)	0.01
Diabetes duration (years)	6.0 (3.0–8.0)	5.0 (3.5–7.0)	6.0 (3.0–8.0)	6.0 (3.0–8.0)	0.59
Antidiabetic treatment					
Lifestyle adjustments	11 (5)	5 (6)	4 (4)	2 (3)	0.55
Metformin	155 (63)	50 (56)	62 (65)	43 (72)	
SU-derivative	9 (4)	3 (3)	4 (4)	2 (3)	
Metformin + SU-derivative	70 (28)	31 (35)	26 (27)	13 (22)	
DM-complications (%)					
Retinopathy	11 (5)	5 (6)	4 (4)	2 (3)	0.80
Neuropathy	32 (13)	11 (12)	11 (12)	10 (17)	0.63
Microalbuminuria	30 (12)	16 (18)	9 (9)	5 (8)	0.13
Antihypertensive drugs (%)					
Diuretics	88 (36)	38 (42)	28 (30)	22 (37)	0.20
Calcium channel blockers	44 (18)	19 (21)	14 (15)	11 (18)	0.53
ACE inhibitors/AT-II receptor antagonists	135 (55)	56 (63)	45 (47)	34 (57)	0.34
Beta blockers	92 (38)	35 (39)	35 (37)	22 (37)	0.95
Statins (%)	205 (84%)	75 (83%)	80 (84%)	50 (83%)	0.99
Cardiovascular disease (%)	67 (27)	23 (26)	32 (33)	12 (20)	0.16
Smoking status					
Current (%)	36 (15)	17 (19)	12 (12)	7 (12)	0.20
Alcohol use (%) ≤2 units/day	216 (89)	75 (83)	84 (88)	66 (92)	0.03
Exposure to sun					
<5 h/week	102 (42)	51 (57)	36 (37.5)	15 (25)	<0.001
5–10 h/week	103 (42)	33 (37)	45 (47.9)	25 (42)	
>10 h/week	40 (16)	5 (6)	15 (15.6)	20 (33)	
Physical activity					
<2 h/week	78 (32)	38 (43)	30 (31)	10 (17)	0.03
2–5 h/week	112 (45)	38 (43)	46 (48)	28 (47)	
>5 h/week	55 (23)	13 (14)	20 (21)	22 (36)	
Season of blood collection					
Spring	24 (10)	10 (11)	11 (11)	3 (5)	0.047
Summer	66 (27)	16 (18)	24 (25)	26 (43)	
Autumn	119 (48)	48 (54)	45 (47)	26 (43)	
Winter	36 (15)	15 (17)	16 (17)	5 (9)	
Body mass index (kg/m^2^)	28.8 ± 4.5	29.9 ± 4.4	28.0 ± 4.6	28.2 ± 3.9	0.009
WTH ratio	0.98 (0.93–1.04)	0.99 (0.92–1.04)	0.97 (0.93–1.04)	1.00 (0.94–1.03)	0.77
AGE-value	2.64 ± 0.6	2.81 ± 0.6	2.63 ± 0.6	2.41 ± 0.5	<0.001
Fasting glucose (mmol/l)	7.5 (6.9–8.2)	7.4 (6.9–8.2)	7.5 (6.9–8.6)	7.7 (6.9–8.4)	0.84
Fasting insulin (mU/l)	14.5 (9.0–21.0)	14.9 (9.5–21.8)	13.8 (8.8–21.0)	14.6 (8.7–20.9)	0.78
HbA_1c_ (mmol/mol)	51 (47–55)	51 (49–54)	52 (45–56)	51 (46–55)	0.96
HbA_1c_ (%)	6.8 (6.5–7.2)	6.8 (6.6–7.1)	6.9 (6.3–7.3)	6.8 (6.4–7.2)	0.96
LDL-cholesterol (mmol/l)	2.5 ± 0.9	2.5 ± 1.0	2.5 ± 1.0	2.4 ± 0.7	0.77
eGFR (ml/min/1.73 m^2^)	81 ± 18	82 ± 20	81 ± 17	82 ± 17	0.94
Calcium (mmol/l)	2.33 ± 0.08	2.32 ± 0.08	2.33 ± 0.08	2.34 ± 0.07	0.22
Albumine (g/l)	40 (38–41)	39 (38–41)	40 (38–41)	40 (39–41)	0.07
Serum 25(OH)D (nmol/l)	60.3 ± 23.4	36.9 ± 8.0	61.5 ± 7.2	92.9 ± 13.4	<0.001
PTH (pmol/l)	5.1 (4.0–6.8)	5.2 (4.4–7.0)	5.4 (4.1–6.9)	4.3 (3.6–5.9)	0.01
AP (U/l)	72.6 ± 20.6	74.6 ± 24.0	70.1 ± 19.3	73.3 ± 16.6	0.38

Data are presented as numbers (%), mean ± SD or median (ICR).

*25(OH)D* 25-hydroxyvitamin D, *ACE* angiotensin-converting enzyme, *AGE* advanced glycation endproduct, *AP* alkaline phosphatase, *AT-II* angiotensin II, *DM* diabetes mellitus, *eGFR* estimated glomerular filtration rate, *PTH* parathyroid hormone, *SU-derivatives* sulphonyl-urea derivatives, *T-chol/HDL ratio* total cholesterol–high density lipoprotein ratio.

Skin autofluorescence values were significantly higher in the vitamin D deficient group compared to the group with a serum 25(OH)D >75 nmol/l (2.81 ± 0.6 versus 2.41 ± 0.5; p < 0.001). Skin autofluorescence significantly raised with increasing age (2.44 ± 0.49 to 2.91 ± 0.61 in patients aged <60 and >70 years, respectively, data not shown). No difference in skin autofluorescence was seen in patients treated with metformin (n = 225) compared to patients without metformin treatment (n = 20) (data not shown).

Linear regression analyses were performed to determine the association between serum 25(OH)D and skin autofluorescence. A significant association between serum 25(OH)D and skin autofluorescence (β = −0.007; p < 0.01) was demonstrated. Confounders for this association were age, ethnicity, season, sun exposure, diabetes duration, presence of cardiovascular disease, eGFR, alkaline phosphatase and LDL cholesterol. No effect of HbA_1c_, sex, smoking behaviour or the use of statins and/or antihypertensive drugs was measured on the association between serum 25(OH)D and skin auto fluorescence. After adjustment for above mentioned confounding risk factors, the association between serum 25(OH)D and skin autofluorescence remained statistically significant (β = −0.006; p < 0.01) (Table 2). For the complete model see Additional file 1.

**Table 2:** Linear regression analysis of serum 25(OH)D (independent variable) and skin auto fluorescence (dependent variable)

	β (95% CI)	SE B	p value
Model 1: crude analysis	−0.007 (−0.010 to −0.004)	0.002	<0.01
Model 2: Model 1 + age, ethnicity, season, smoking, BMI	−0.007 (−0.010 to −0.004)	0.002	<0.01
Model 3: Model 2 + sun exposure, diabetes duration, CVD, HbA_1c_, eGFR, AF, LDL cholesterol	−0.006 (−0.009 to −0.003)	0.002	<0.01

*25(OH)D* 25-hydroxyvitamin D, *AGE* advanced glycation end product, *AP* alkaline phosphatase, *BMI* body mass index, *CVD* cardiovascular disease, *LDL* low density lipoprotein.

**Figure 1:**
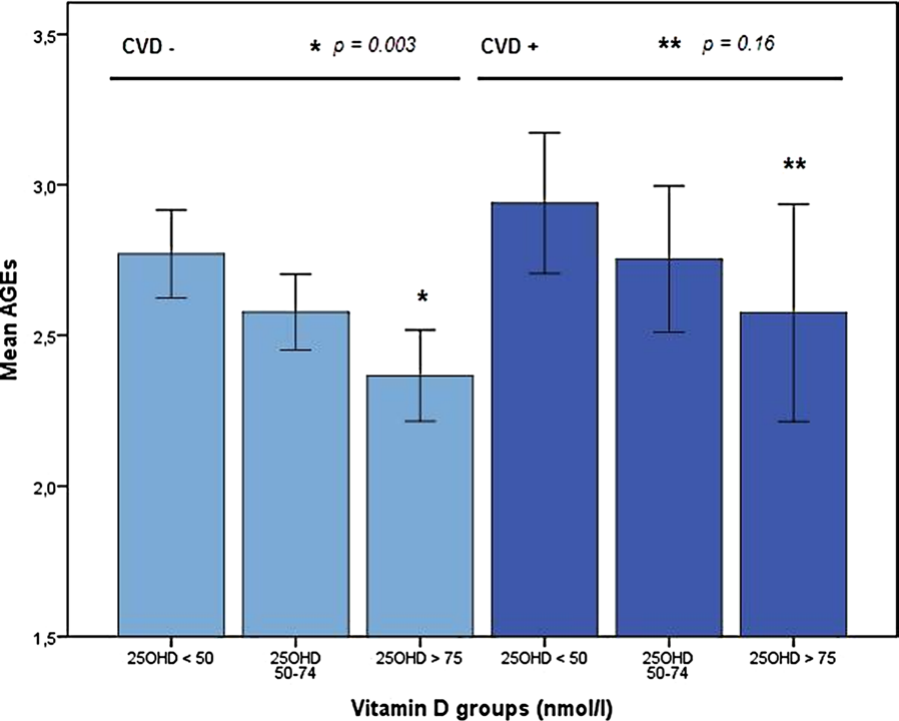
Mean skin autofluorescence in vitamin D subgroups stratified by cardiovascular disease. *25OHD* 25-hydroxyvitamin D, *AGE* advanced glycation endproducts, *CVD* cardiovascular disease.

In patients with previous cardiovascular disease (n = 67) mean skin autofluorescence was significantly higher compared to patients without cardiovascular disease (n = 178) (AGEs: 2.79 ± 0.57 and 2.59 ± 0.62; *p* = 0.02, respectively). In patients without previous cardiovascular disease mean skin autofluorescence was significantly higher in patients with a serum 25(OH)D level <50 nmol/l compared to patients with a serum 25(OH)D level >75 nmol/l (2.77 ± 0.60 and 2.37 ± 0.52; *p* = 0.003, respectively). This result was also seen in patients with cardiovascular disease, although this difference was not statistically significant (2.94 ± 0.54, and 2.58 ± 0.57 in patients with a serum 25(OH)D level <50 and >75 nmol/l; *p* = 0.16, respectively) (Figure 1).

### Longitudinal analysis

210 out of 245 (85%) patients completed the trial and conducted a skin AGE measurement after 6 months. The majority of the excluded patients throughout the study had changed their hypoglycemic agents on own initiative or due to an HbA_1c_ level >69 mmol/mol (n = 19), two patients had a serum 25(OH)D <15 nmol/l, one patient suffered from new onset urolithiasis, and eight patients did not show at their last visit. Serum 25(OH)D raised from 60.8 to 103.6 nmol/l in the vitamin D group (n = 107) (*p* = < 0.01) and decreased from 61.0 to 60.3 nmol/l in the placebo group (n = 103) (*p* = 0.78). Mean skin autofluorescence significantly increased (2.63 ± 0.53 to 2.74 ± 0.56; *p* = 0.02) in the placebo group, whereas in the vitamin D group mean skin autofluorescence increased less profoundly which was not significant (2.67 ± 0.64 to 2.73 ± 0.69; *p* = 0.19). Comparing the change in mean skin autofluorescence from baseline to 6 months, no significant difference was found between both groups (β −0.05, 95% CI −0.17 to 0.08, p = 0.47). No significant change over time in HbA_1c_ was seen in the intervention group whereas mean BMI increased significantly (Table 3).

**Table 3:** Comparison of the main outcomes before and after intervention between both groups

Variable	Vitamin D group (n = 107)		Placebo group (n = 103)		β (95% CIs)	p value
	0 month	6 months	0 month	6 months		
Serum 25(OH)D (nmol/l)	60.8 ± 23.0	103.6 ± 25.7	61.0 ± 23.4	60.3 ± 26.5	42.5 (35.8–49.6)	<0.01
AGE-value	2.67 ± 0.64	2.73 ± 0.69	2.63 ± 0.53	2.74 ± 0.56	−0.05 (−0.17 to 0.08)	0.47
BMI (kg/m^2^)	28.8 ± 4.8	29.1 ± 4.4	28.7 ± 4.7	28.8 ± 4.8	0.23 (0.01–0.46)	0.04
HbA_1c (_mmol/mol)	51.1 ± 6.0	50.0 ± 6.3	50.3 ± 5.3	49.4 ± 6.2	1.2 (−0.1 to 2.6)	0.07

*25(OH)D* 25-hydroxyvitamin D, *AGE* advanced glycation end product, *BMI* body mass index.

## Discussion

We found a significant inverse association between serum 25(OH)D and skin autofluorescence independent of major confounders, including age, season, diabetes duration and renal function in a group of patients with relatively tight controlled T2DM. For each 10 nmol/l increment in serum 25(OH)D level skin autofluorescence decreases with 0.06 in patients with T2DM. Patients with a serum 25(OH)D <50 nmol/l had a significantly higher skin autofluorescence compared to patients with an adequate serum 25(OH)D level >75 nmol/l (2.81 ± 0.6 and 2.41 ± 0.5, respectively). Six months of vitamin D supplementation did not alter the amount of skin auto fluorescence significantly compared to the placebo group. However, the duration of the intervention could be too short for this outcome measure, as skin AGEs have a mean half live of 10–15 years [35].

A possible underlying mechanism for the association found between skin AGEs and vitamin D status, could be a reduction of oxidative stress by vitamin D and thereby a decrease in the formation of AGEs and pro-inflammatory cytokines that mediate microvascular and macrovascular complications in T2DM. A recent in vitro study demonstrated a decrease of cytokine-mediated endothelial inflammation after addition of calcitriol, supporting the concept that calcitriol may act as a vascular protective agent counteracting the probable deleterious actions of AGEs on endothelial cell activities [13, 36]. Moreover, several studies have shown protective effects of vitamin D on vascular wall function and cell membranes by maintaining steady levels of certain intracellular antioxidants, by reducing lipid peroxidation and by reducing the overproduction of reactive oxygen species [37]. A recent study performed by Stürmer et al. [38] among 119 healthy en 27 hypertensive non-diabetic participants with a mean serum 25(OH)D 56.2 ± 22.2 nmol/l, revealed no association between vitamin D status and skin autofluorescence.

Our results regarding the significantly higher skin autofluorescence found in patients with previous cardiovascular disease compared to patients without cardiovascular disease (mean skin autofluorescence: 2.79 ± 0.57 versus 2.59 ± 0.62) are in line with previous studies which demonstrated mean skin autofluorescence values of 2.57 in patients with T2DM without diabetes-related complications, and skin AGEs of 3.12, 2.91 and 2.71 in patients with T2DM and both micro- and macrovascular complications, solely macrovascular complications and solely microvascular complications, respectively [7].

Interestingly, the patients in the highest vitamin D group in our study had a mean skin autofluorescence comparable with the non-diabetic population demonstrated in a study by Koetsier et al. [39]. The authors generated a formula for calculating the mean skin autofluorescence in the general population without diabetes mellitus: 0.024 × age + 0.83. When this formula is applied to our study population with a mean age of 67 years, the calculated mean skin autofluorescence is 2.44. In our study results the patients with T2DM in the highest vitamin D group had a lower mean autofluorescence level: 2.41 ± 0.5. This could imply a protective effect of vitamin D on the formation and accumulation of AGEs in patients with T2DM. However, an important limitation in our study is that we measured the accumulated AGEs in the skin using an AGE-reader, and other known AGE types, such as circulating AGEs and AGEs without fluorescent properties could have provided different results within the vitamin D subgroups. In addition from this point of view the association between vitamin D status and skin autofluorescence found in our study could be explained by the fact that in theory cutaneous AGE accumulation could hinder the photoconversion of the provitamin D into vitamin D. Nevertheless, our results show that vitamin D sufficiency is mainly found in patients with T2DM without high cardiovascular risk profile as indicated by the age-adjusted AGE levels within the normal range. Furthermore, we plan to conduct future analyses with the stored serum of all patients to measure circulating AGEs.

In contrast to previous studies, smoking was not correlated to skin autofluorescence in our study. This may be due to the small group of smokers in our study population (n = 36; 15%). HbA_1c_, which is also an end product of glycation, was not associated with skin autofluorescence. This is not surprisingly as earlier research demonstrated only a weak effect between skin autofluorescence and HbA_1c_. A logical explanation is that HbA_1c_ represents AGE accumulation over a short period of approximately 6–8 weeks, while skin AGEs accumulate over 10–15 years [35].

In conclusion, this preliminary report demonstrates an independent association between vitamin D status and skin autofluorescence in patients with tight controlled T2DM. A short intervention of vitamin D supplementation, however did not have any effect on the amount of skin autofluorescence. Future research should focus on measuring circulating AGEs after a longer follow-up and treatment with vitamin D.

## Additional file

Additional file 1: Linear regression analysis of serum 25 (OH)D as independent variable and skin auto fluorescence as dependent variable. Model 1: crude analysis. Model 2: as model 1 + age, ethnicity, season, smoking, and BMI. Model 3: as model 2 + sun exposure, diabetes duration, CVD, HbA1c, eGFR, AF (this should be AP), and LDL cholesterol. *25(OH)D* 25-hydroxyvitamin D, *AGE* advanced glycation end product, *AP* alkaline phosphatase, *BMI* body mass index, *CVD* cardiovascular disease, *LDL* low density lipoprotein.

## Abbreviations

25(OH)D: 25-hydroxyvitamin D
AP: alkaline phosphatase
AST: aspartate transaminase
CVD: cardiovascular disease
MAP: mean arterial pressure
PTH: parathyroid hormone
VDR: vitamin D receptor
